# The Cognitive Control Model of Work-related Flow

**DOI:** 10.3389/fpsyg.2023.1174152

**Published:** 2023-06-13

**Authors:** Jared Weintraub, Kevin P. Nolan, Aditi Rabindra Sachdev

**Affiliations:** Psychology Department, Hofstra University, Hempstead, NY, United States

**Keywords:** flow, mindfulness, grit, metacognition, performance, burnout, engagement

## Abstract

Although several models of flow have been proposed that include environmental and trait-based antecedents of the state, elements of cognitive control that enable workers to experience flow and its subsequent outcomes at work have largely been overlooked. This research proposes and provides empirical support for the “Cognitive Control Model of Work-related Flow,” which integrates antecedents of flow at work related to the ability to focus concentration of cognitive resources toward experiencing flow at work. Along with flow at work, the model includes the antecedents of grit, flow metacognition, and mindfulness at work and the outcomes of work performance, engagement, and burnout. Findings across three studies (a cross-sectional, a time-lagged, and a one-day experience sampling method study) utilizing MTurk participants provided support for the model, as grit, mindfulness, and flow metacognition predicted flow, and flow predicted subjective performance, engagement, and burnout. Theoretical implications and the potential for developing flow interventions at work are discussed.

## Introduction

“Flow”—characterized by “an engrossing and enjoyable state of mind that occurs when people feel optimally challenged and are fully absorbed in their current activity” (Debus et al., 2014, p. 713)—has been associated with fundamental work outcomes such as increased performance (Demerouti, 2006), reduced burnout (Lavigne et al., 2012), and increased engagement (Fraga and Moneta, 2016; Medhurst and Albrecht, 2016). Although existing literature has identified several potential antecedents of flow (Eisenberger et al., 2005; Guo and Poole, 2009; Ullén et al., 2012; Crust and Swann, 2013; Culbertson et al., 2015; Fong et al., 2015), most focus on hereditary individual differences (i.e., personality and trait intrinsic motivation; Ullén et al., 2016) or situational variables such as the environment (i.e., perceived positive work environment and a culture of feedback) and characteristics of one's job (i.e., autonomy and task variety; Demerouti and Mäkikangas, 2017). Although understanding the dispositional and situational factors that facilitate flow at work is necessary for a theoretical understanding of the construct and for the creation of “flow-friendly” organizational policies and procedures, these environmental antecedents are often difficult to control or modify, and hereditary differences are not readily amenable to change interventions.

**Figure 1:**
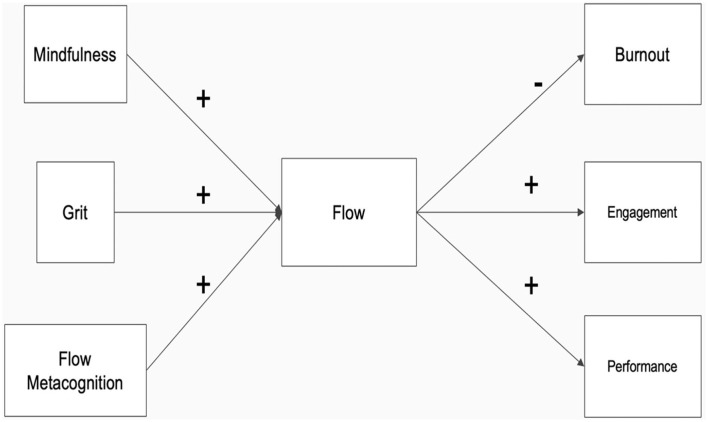
Proposed Cognitive Control Model of Work-related Flow. Direct effects of mindfulness, grit, and flow metacognition on burnout, engagement, and performance were removed from the figure for parsimony but were included in analyses.

In this study, we offer the “Cognitive Control Model of Work-related Flow” (CCMWF; Figure 1) as a supplement to existing knowledge about the situational and dispositional factors that influence flow at work. The CCMWF builds upon the tenets of the conservation of resources theory (Hobfoll, 1989), as well as popular theories of motivation (e.g., Vroom, 1964), behavior (Ajzen, 1988), and decision-making (e.g., Tversky and Kahneman, 1992), to present a collection of antecedents that include: workers' beliefs about the utility of experiencing flow for job performance (i.e., flow metacognition), focus of cognitive resources on the pursuit of flow (i.e., mindfulness), and continued pursuit despite setbacks to entry (i.e., grit).

Given that flow is a volitional state that requires the expenditure of personal resources to enter and sustain (Keller and Bless, 2008; Demerouti and Mäkikangas, 2017), the CCMWF purports that the more workers believe that flow benefits their job performance, focus their cognitive resources on the pursuit of the state, and maintain that focus despite initial setbacks to entry, the more frequently they will experience flow at work and its associated benefits. Support for the model is provided via three empirical research studies (cross-sectional, time-lagged, 1-day experience sampling method). Findings serve to advance the theory of work-related flow via further explication of its nomological network, better understanding the mechanisms that motivate and enable individuals to pursue flow at work, and affording insights into how pragmatic interventions aimed at facilitating flow may be designed to increase the state for those wanting to increase it by their own volition—especially when modifications to the work environment are improbable or impractical. Moving ahead, we will further explore the construct of flow, the outcomes associated with flow at work, the antecedents of flow at work, and the theoretical and practical rationale for the development of the proposed model.

### Flow at work

Csikszentmihalyi (1975) describes flow as, “the holistic sensation that people feel when they act with total involvement” (p. 36). Decades of research have supported the notion that flow is associated with higher wellbeing (Tse et al., 2020), creativity (Schutte and Malouff, 2020), and performance across a multitude of domains (Bakker et al., 2011; Rutrecht et al., 2021; Weintraub et al., 2021). In the workplace, in particular, flow has been shown to influence important behaviors and psychological states such as greater communication effectiveness (Webster et al., 1993), organizational spontaneity (Eisenberger et al., 2005), creativity (Yan et al., 2013), engagement, stress, and performance (Weintraub et al., 2021).

However, it is important to note constraints inherent in the workplace can fundamentally change how flow is pursued and experienced at work (Bakker, 2008; Bakker and van Woerkom, 2017; Fullagar and Delle Fave, 2017). For example, in his seminal book, Csikszentmihalyi (1975) suggests that the state is most likely to be experienced when a person *believes* that they have the skills necessary to complete a task that they perceive to be of a moderate-to-high level of difficulty. While people can often choose tasks for themselves outside of the work domain which are challenging, yet not overwhelming, employees often lack the autonomy to choose the tasks at work they are required to focus on, and these tasks may induce boredom or anxiety, which have been shown to inhibit flow at work (Demerouti, 2006; Nielsen and Cleal, 2010).

In general, research has tended to highlight the importance of antecedents related to personality (Tse et al., 2020), genetics (Mosing et al., 2012), and one's environment (Joo et al., 2011) for fostering the state. With regard to antecedents of flow at work specifically, using the lenses of job demands-resources theory (JD-R; Bakker and Demerouti, 2014) and the job characteristics model (Hackman and Oldham, 1980), studies have found support for the idea that aspects of the work environment, such as different types of work and working conditions, may innately help or hinder employees' ability to experience flow on the job by providing clear goals, intrinsic motivation toward the task at hand, and the balance of challenge and skills necessary for experiencing the state (Demerouti, 2006; Nielsen and Cleal, 2010; Fagerlind et al., 2013; Maeran and Cangiano, 2013; Zito et al., 2016; Spurlin and Csikszentmihalyi, 2017). Demerouti and Mäkikangas (2017) suggest that hindering job demands can reduce flow, while flow is most frequently experienced when jobs have a high level of job resources, along with high (yet manageable) job demands. Moreover, in line with the Job Characteristics Model (Hackman and Oldham, 1980), research has identified role clarity, skill variety, task significance, feedback, and autonomy as aspects of jobs that may facilitate flow at work (Fullagar and Kelloway, 2009; Steele and Fullagar, 2009; Nielsen and Cleal, 2010; Maeran and Cangiano, 2013; Fullagar et al., 2017).

These studies provide a foundation for organizations to design work and environments at the macro level which can facilitate flow, and potentially allow for the development of assessments and recruitment strategies for finding workers who are more likely to experience flow at work. However, despite the early focus on flow as a state driven by individual beliefs and motivation (Csikszentmihalyi, 1975; Keller and Bless, 2008), there is little empirical examination of what motivates the directing of resources toward experiencing flow or examining how individuals may purposefully experience flow at work—despite environmental or dispositional constraints. Namely, if there is little flexibility in the type of work employees can choose to do, or if organizations have already hired employees with dispositional traits that are not necessarily conducive to the state, little guidance can be found in the literature as to what can be done to promote flow within their workforce. Additionally, if a worker is in a role that does not naturally provide job characteristics, resources, or demands conducive to experiencing flow at work, there are few empirical sources they can consult for direction as to how they can increase the state by their own volition. This gap in the literature leaves many open questions about the role that these additional factors play in facilitating flow at work, and how they might interact with the more traditionally studied variables previously discussed. More specifically, there is potentially a slew of flow drivers which have been mostly unexplored, and little is known about what motivates workers to pursue the self-directed state in the workplace.

### The Cognitive Control Model of Work-related Flow

The Cognitive Control Model of Work-related Flow is built on the notion that cognitive resources are limited, flow is volitional, requires effort (Keller and Bless, 2008; Harris et al., 2017a), and that there is a need for a better understanding of the factors that motivate and enable people to experience flow at work (Bakker and van Woerkom, 2017; Demerouti and Mäkikangas, 2017; Fullagar and Delle Fave, 2017). More specifically, the proposed model expands, integrates, and answers the call for extant research related to the key antecedents of flow metacognition, grit, and mindfulness (Wilson and Moneta, 2016; Smith et al., 2020; Marty-Dugas et al., 2021). The CCMWF proposes that these variables fit naturally together into a single model, such that they collectively motivate employees to pursue and persistently allocate cognitive resources toward flow. The antecedents featured in the model also answer the call for the exploration of variables influencing flow that are amenable to practical intervention (Weintraub et al., 2021). In addition, the model serves to further contribute to the nomological network and development of the theory of work-related flow by including the outcomes of engagement, burnout, and performance, which have been associated with flow in the past, but require more rigorous examination and support (Fullagar et al., 2017). Therefore, the CCMWF proposes an integrated model where the antecedents of flow metacognition, grit, and mindfulness indirectly influence these key work outcomes through the prevalence of flow experiences.

### Flow metacognition

Although flow experiences are commonly associated with a variety of benefits that include wellbeing, personal resources, and job performance (Llorens and Salanova, 2017), research also suggests that there are potential downsides to experiencing the state such as reduced self-awareness and awareness of time, obscured perceptions of risk and ability, and the potential to become mired in actions that are incongruent with short- and long-term goal accomplishment (Rheinberg, 1991; Guptill, 2012; Schüler, 2012). Accordingly, in their investigations of people's metacognitive beliefs about flow states, Wilson and Moneta (2016) found significant variance in perceptions of its usefulness for performing tasks—including those related to work.

Research on flow metacognitions is in the nascent stages of development. Nevertheless, examining people's beliefs about the usefulness of the state for performing various behaviors is one potentially fruitful answer to the call for a greater understanding of individual differences in flow propensity, especially those relating to goal pursuits, that are amenable to intervention. The CCMWF focuses on workers' beliefs about the usefulness of flow for job performance, as effective job performance is a fundamental goal that workers are generally motivated to pursue. Consistent with the tenets of the theory of planned behavior (Ajzen, 1988) and expectancy theory (Vroom, 1964), the CCMWF purports that workers will be more likely to engage in behaviors that facilitate entry into the flow state when they believe that doing so will benefit their job performance. With flow being a self-directed, volitional state, workers with greater beliefs about its usefulness for job performance are expected to be more likely to dedicate resources toward the pursuit of the state. Metacognitive beliefs about the usefulness of flow for job performance are, therefore, hypothesized to have a positive influence on the frequency with which workers experience the state on the job. Therefore, the current research proposes the following hypothesis:

*Hypothesis 1:* Metacognitive beliefs about the utility of work-related flow for job performance will positively predict the frequency with which workers experience flow on the job.

### Mindfulness

Flow is a chaotic state in that it may be experienced in one moment, and then gone in the next, seemingly at random (Ceja and Navarro, 2009, 2012). Relatedly, Dust (2015) suggests that throughout the workday, our minds are continually cycling through multiple states, which may help or hinder our ability to perform well on the job. One such state which has gained enormous popularity in American culture is mindfulness (Hyland et al., 2015). Kabat-Zinn (1994) defines mindfulness as, “paying attention in a particular way: on purpose, in the present moment, and nonjudgmentally” (p. 4). Dane (2011) suggests that flow and mindfulness are similarly dependent upon high orientation to the present moment, but they are distinct, in that flow requires a narrow concentration on the task at hand while mindfulness has a broader attentional breadth. Subsequent research has found support for mindfulness positively influencing flow by increasing self-regulation of attention and reducing anxiety (Aherne et al., 2011; Scott-Hamilton et al., 2016; Lambert and Csikszentmihalyi, 2019; Marty-Dugas et al., 2021). As such, it is proposed that mindfulness be included in the CCMWF given its ability to help workers concentrate and reduce perceptions of anxiety to promote or restore the balance of challenge and skill required to experience flow at work.

Recently, Hafenbrack and Vohs (2018) found that mindfulness may enable people to detach from stressors, which can improve task focus. Given that hindrance stress has been shown to negatively affect flow (Oortmerssen et al., 2019), and that a tenet of flow theory is that flow and anxiety are incompatible states (Csikszentmihalyi, 1975), the ability of mindfulness to reduce anxiety and enable task focus (Brunyé et al., 2013; Grégoire and Lachance, 2015) is proposed to facilitate flow at work. Support has been found for this assertion in previous research by Scott-Hamilton and Schutte (2016), which found that a mindfulness intervention reduced anxiety in cyclists and increased flow experience.

Additionally, Good et al. (2016) proposed that mindfulness can help people concentrate and keep their attention from being hijacked by distractions. Attentional control is a resource that is key to the experience of flow at work (Harris et al., 2017a,b), and concentration on the task at hand is a key tenet of flow theory (Csikszentmihalyi, 1975). Therefore, it is proposed that state mindfulness fosters this key resource and contributes to the ability of individuals to experience flow at work by enabling workers to allocate their cognitive resources toward the state. However, the relationship between mindfulness and flow is understudied (Marty-Dugas et al., 2020), and to date, there is a particular dearth of empirical research investigating this relationship in the work domain.

Given the negative relationships between flow, and burnout and anxiety (Lavigne et al., 2012; Fullagar et al., 2013; Mosing et al., 2018), the ability of state mindfulness to mitigate these constructs (Scott-Hamilton and Schutte, 2016; Lomas et al., 2018), as well as mindfulness' ability to increase attentional control (Bhayee et al., 2016), state mindfulness is proposed to facilitate flow. As an answer to the call for more research investigating mindfulness and flow (Marty-Dugas et al., 2020), this research aims to integrate state mindfulness into the CCMWF as an antecedent that enables workers to allocate their cognitive resources toward experiencing flow at work. Therefore, the following hypothesis is proposed:

*Hypothesis 2:* Mindfulness will positively predict the frequency with which workers experience flow on the job

### Grit

The proposed model is rooted in the idea that flow is a volitional and effortful state (Keller and Bless, 2008; Harris et al., 2017a) that requires attentional control (Harris et al., 2017b) and commensurate levels of challenge and skill (Fong et al., 2015). Given that the workplace is often full of distractions and setbacks, which can make expending this effort and attentional control more difficult for workers to experience flow, we propose grit as an individual difference that allows employees to experience flow at work through skill development and persistent effort. Introduced by Duckworth et al. (2007), grit is defined as perseverance and passion for long-term goals. Grit focuses on individuals who have higher order goals and pursue them over the years despite setbacks (Duckworth et al., 2007). The work of Duckworth et al. (2007) and subsequent research correlate measures of grit and individual performance, above and beyond self-control and conscientiousness (Duckworth and Gross, 2014; Duckworth and Seligman, 2017).

Flow and grit are directly related in the context of students studying music (Miksza and Tan, 2015; Miksza et al., 2016) and students' spelling bee performance (Von Culin et al., 2014). Only recently, Smith et al. (2020) proposed and found evidence for the notion that gritty individuals are better able to avoid distractions and direct their attention toward flow. Both Duckworth (2016) and Smith et al. (2020) explicitly call for more research to further validate and examine the nuances of this relationship. As an answer to this gap in the literature, the current research seeks to examine the relationship between flow and grit within the context of the CCMWF. It proposes that grit contributes to the ability of employees to experience flow at work through skill development and persistent effort, where developing the skills required to meet work demands results in the balance of challenge and skill required for flow, and persistent effort allows for the required cognitive control to be consistently focused toward experiencing flow at work despite distractions and setbacks.

With regard to skill development, Duckworth et al. (2011) found that people high in grit are more likely to engage in deliberate practice (Ericsson et al., 1993). Deliberate practice has four main components: well-defined goals, challenge level exceeding one's skill, immediate feedback, and a repetitive focus on error correction (Ericsson et al., 1993; Duckworth, 2016; Eskreis-Winkler et al., 2016). These components have clear parallels with the flow; both focus on clear goals that are challenging yet achievable, require feedback, and corrections must be made for each to be maintained. Additionally, researchers suggest that to continuously experience flow, one must consistently challenge themselves as their skills are developed over time (Csikszentmihalyi, 1990; Ceja and Navarro, 2012; Oortmerssen et al., 2019), and that deliberate practice is necessary for fostering the skills required to meet these challenges (Von Culin et al., 2014). While gritty individuals focus on long-term goals, it is proposed that their consistent focus on error correction, and the immediate feedback associated with deliberate practice helps to foster flow in the short-term. For example, if an employee is facing immediate setbacks which are hindering their ability to enter flow, a gritty individual is proposed to be more likely to correct errors and seek immediate feedback to overcome these obstacles and experience flow. In line with this assertion, research on professional artists has shown that they participate in rituals every day that they believe will help them to experience and maintain flow to be creative (Currey, 2013).

A second way in which grit is proposed to influence flow is through persistent focusing of cognitive resources. Given that flow is difficult to accomplish and requires effort (Harris et al., 2017a), it is proposed that “gritty” individuals who persevere despite setbacks will be more likely to overcome obstacles to flow, and thus, experience the intrinsic reward inherent in the state. Additionally, it is proposed that this feeling of intrinsic reward acts as a positive reinforcement for the sustained effort individuals have exerted over time and increases the likelihood that they will continue to intentionally allocate their cognitive resources toward their efforts to experience flow. To this end, Von Culin et al. (2014) found that the motivation to engage in flow-producing activities facilitates sustained effort over time toward long-term goals.

Given that flow requires a balance of challenge and skill (Nakamura and Csikszentmihalyi, 2009) and that “increasing one's skill level is necessary in order to accommodate increasing challenges if one is to experience, or remain in a flow state” (Lambert and Csikszentmihalyi, 2019, p. 10), it is proposed that grit's role in skill development will facilitate flow experience. Additionally, given that experiencing flow requires effort (Harris et al., 2017a) and sustained attention (Harris et al., 2017b; Marty-Dugas et al., 2020), the perseverance of gritty individuals is proposed to help employees achieve flow at work by allowing for the persistent exertion of cognitive resources toward entering the state. As such, the current research seeks to integrate grit into the CCMWF as an individual antecedent, which enables individuals to develop the skills and have the perseverance required to allocate their cognitive resources toward experiencing flow at work continuously. Therefore, the following hypothesis is proposed:

*Hypothesis 3:* Psychological grit will positively predict the frequency with which workers report experiencing flow on the job.

### Integration of outcomes of flow at work

To gain a more holistic understanding of the nomological network of flow at work, it is crucial to examine both the direct effects of the antecedents composing the CCMWF on personal and organizational outcomes, as well as their indirect effects through flow. Flow at work has been positively associated with outcomes of wellbeing, such as job satisfaction (Maeran and Cangiano, 2013), positive mood (Fullagar and Kelloway, 2009), commitment (Salanova et al., 2005), and subjective wellbeing (Bloch, 2002), and negatively associated with burnout and anxiety (Lavigne et al., 2012; Fullagar et al., 2013; Mosing et al., 2018). Additionally, flow has been associated with engagement (Fraga and Moneta, 2016; Medhurst and Albrecht, 2016; Weintraub et al., 2021), service quality in customer service personnel (Kuo and Ho, 2010), and performance (Demerouti, 2006; Bakker, 2008; Weintraub et al., 2021).

These outcomes are especially important in that, according to Gallup (2022), ~80% of global workers are not engaged at their places of work, which is estimated to cost the global economy $7.8 Trillion in lost productivity annually or about 11% of GDP globally. Meanwhile, the World Health Organization recently declared burnout (an affective response to stress including emotional exhaustion, physical fatigue, and cognitive weariness; Schaufeli and Buunk, 2003; Shirom, 2003; Melamed et al., 2006) an “occupational phenomenon” (World Health Organization, 2019), which has been shown to increase the risk of suicide and is on the rise around the globe (Shirom, 2011; Sigsbee and Bernat, 2014). Gallup's (2022) most recent *Stat of the Global Workplace* Report also found stress to reach an all-time high in the global workforce for the second year in a row. Additionally, performance can be considered one of the most important professional outcomes because it is a key driver of employment status (e.g., being hired or fired, promoted or demoted; Equal Employment Opportunity Commission, 1978; Civil Rights Act, 1991). Given the importance and timeliness of these constructs, and their relationships with flow, researchers and practitioners need to understand how flow can be induced as a potential way to mitigate the downsides of these outcomes while enhancing their benefits.

Like flow, mindfulness and grit have each been associated with engagement, burnout, and performance (Duckworth et al., 2009; Suzuk et al., 2015; Good et al., 2016; Walker et al., 2016). However, research on flow metacognition is still in its early phases, and these proposed antecedents have yet to be integrated into a holistic model of flow. Given that grit, flow metacognition, and mindfulness are proposed to lead to flow, which then predicts the outcomes of engagement, burnout, and performance, the proposed model includes flow as a mediator between its antecedents and the outcomes of interest. As such, the current research seeks to integrate distal calls for research exploring the relationship between the proposed antecedents and flow by integrating them into the CCMWF to further develop a nomological network and advance the theory of work-related flow.

While several models of flow have been proposed in the past (Moneta, 2012; Bakker and van Woerkom, 2017; Demerouti and Mäkikangas, 2017), most focus on aspects of work that may be difficult for individuals to control. Moreover, none have been tested that focus on the cognitive control aspects of flow discussed, nor have empirical investigations fully integrate these antecedents, flow, and key outcomes of the workplace. The proposed model (Figure 1) contributes to the theory of the construct of flow at work and its nomological network, in that support for the model would provide a new understanding of how constructs related to cognitive control influence flow, and subsequently, engagement, burnout, and performance. Furthermore, support for this model lays the foundation for researchers and practitioners to test new interventions that individuals may be able to implement on their own, even if they are unable to influence their work environment and the characteristics of their jobs in a meaningful way. Given the above, the following hypotheses are proposed:

*Hypothesis 4a,b,c:* Flow mediates the relationships between flow metacognition (a), mindfulness (b), grit (c), and performance.
*Hypothesis 5a,b,c:* Flow mediates the relationships between flow metacognition (a), mindfulness (b), grit (c), and engagement.
*Hypothesis 6a,b,c:* Flow mediates the relationships between flow metacognition (a), mindfulness (b), grit (c), and burnout.

## Method

### Overview of studies

The proposed Cognitive Control Model of Work-Related Flow (Figure 1) provides a framework through which to examine the nomological network of flow in the workplace with a focus on one's motivation and ability to exercise cognitive control toward experiencing flow and its positive outcomes at work. To test the validity of the model sufficiently, a 3-study research agenda was employed to develop the proper measurement tools and test the model at both the between and within levels. Study 1 was a cross-sectional study conducted to provide validation for a Flow Metacognition scale specifically aimed at measuring if people vary in the extent to which they perceive flow as useful for the jobs they perform, and to examine covariance with the prevalence with which flow is experienced at work. Study 2 utilized a time-lagged approach to examine the relationships hypothesized by the CCMWF using trait-level measures. Study 3 utilized a one-day experience sampling methodology to further examine the relationships hypothesized by the CCMWF using state measures of mindfulness and flow to analyze within-person effects and provide further support for the CCMWF. Together, these three studies provide a robust test of the CCMWF by first providing initial validation of a new scale for measuring flow metacognition and then testing the broader model at both the trait and state levels.

### Study 1 purpose

Limited research has examined metacognitive beliefs about flow. As it pertains to work, research has solely focused on workers' cognitions about the state for single, non-descript activities (Wilson and Moneta, 2016). This study examines variance in workers' metacognitive beliefs about the utility of work-related flow for job performance (in general) while testing the psychometric properties of a newly developed scale; and examines covariance among these measures, flow prevalence experienced on the job, and several work attributes that may influence metacognitive beliefs about the utility of the state. These attributes include the level of social interaction and RIASEC vocational interests (Holland, 1985) that characterize work. Insights are provided about variance in metacognitive beliefs about the utility of work-related flow for job performance using a diverse sample of workers, and understanding of the construct is advanced through examining relationships in its nomological network.

Scholars have proposed that flow might not be useful for all types of job performance given the state is associated with reduced self-awareness and awareness of time, obscured perceptions of risk and ability, and the potential to mire workers in actions that are inconsistent with short- and long-term goal accomplishment (Schüler, 2012). The loss of self-awareness and exclusive concentration on the task at hand that characterize flow can produce social conflict (Schüler, 2012). Likewise, external regulations can disrupt flow experiences (Keller and Bless, 2008), and the social conventions of work have been suggested to mitigate positive outcomes associated with experiencing the state (Csikszentmihalyi and LeFevre, 1989). As such, the level of social interaction characterizing jobs is examined as a potential antecedent of metacognitive beliefs about the utility of work-related flow for job performance.

Holland's theory of vocational personalities and work environments (a.k.a., the RIASEC theory) is the most widely used and researched model of occupational attributes (Holland, 1997; Wille and De Fruyt, 2014). Work environments that fulfill artistic vocational interests commonly involve working with designs and patterns in ways that are creative and emotionally expressive (Holland, 1997). Research suggests that flow is positively related to creative experiences (Llorens and Salanova, 2017); this relationship is facilitated by a combination of the state's positive affective components (e.g., positive mood and enjoyment) and the heightened concentration that results from an optimal balance between high challenge and high skill (Fullagar et al., 2017). Work environments that fulfill conventional vocational interests commonly involve predictable job demands that are ordered and have specified standards (Holland, 1997). Research suggests that flow occurs more often and for longer durations when activities have structured task conditions that include clear goals and immediate feedback (Csikszentmihalyi, 1990). Similarly, research has found that flow is perceived as more rewarding when experienced during work that follows a pattern (Csikszentmihalyi, 1975). As such, study 1 will conduct exploratory research examining the extent to which jobs fulfill artistic and conventional vocational interests and are also potential antecedents of metacognitive beliefs about the utility of work-related flow for job performance. The findings of this study will help to further our understanding of the nomological network of flow at work, while also providing more support for the validity of a new tool for measuring flow metacognition that can be utilized to test the proposed hypotheses in subsequent studies.

### Study 1 participants and procedure

A sample of *n* = 552 workers in the United States who work at least 25 h per week and were 18 years of age or older was recruited through Amazon Mechanical Turk (MTurk), a crowdsourcing website that research suggests is a viable source of high-quality data for the social sciences (Casler et al., 2013). Participants who did not complete the full survey provided incorrect responses to reading prompts embedded in the survey, exhibited careless responses, reported being professional survey takers, and/or completed the survey in an unreasonably short amount of time were removed from the data set. The final sample included *n* = 393 participants from 221 unique occupations, who were majority male (56%) and White (76%) with a mean age of 36.71 years. Most participants reported working for for-profit organizations (76%), having ~17 years of work experience and 6 years of job tenure in their current positions. Support for the representativeness of the sample is provided by U.S. Bureau of Labor Statistics (2016) data, which suggests that the American workforce is likewise majority male (53%), Caucasian (64%), and working in the for-profit sector (76%). Participants first read a description of the flow state from the Flow Questionnaire (Csikszentmihalyi and Csikszentmihalyi, 1988; Appendix A). They then completed the self-report measures in counterbalanced order to avoid issues relating to common method bias (Podsakoff et al., 2003).

### Study 1 measures

#### Flow metacognition

A 6-item scale based on the “Beliefs that Flow Fosters Achievement” dimension of Wilson and Moneta's (2016) Flow Metacognition scale was created by two subject matter experts for purposes of being tested in study 1 to measure workers' metacognitive beliefs about the usefulness of work-related flow. Rather than focusing on a single activity that is “most representative of the flow experience” (p. 226), the items on this measure were revised to assess the metacognitive beliefs about the utility of flow for job performance in general. For example, the item “Flow has a positive effect on the activity” was modified to read “Regularly experiencing flow would enhance your overall job performance.” Responses were reported using a 5-point (1 = strongly disagree, 5 = strongly agree) Likert scale. An initial confirmatory factor analysis suggested a moderately acceptable fit between the measurement model and the data collected from the sample, $\chi_{\left(9\right)}^{2}$ = 70.14, *p* < 0.01, *GFI* = 0.94, *CFI* = 0.97, *RMR* = 0.03, *RMSEA* = 0.13. Following modification indices, a second confirmatory factor analysis was conducted on a 4-item version of the measure. Results indicated markedly improved fit, $\chi_{\left(2\right)}^{2}$ = 0.50, *p* < 0.77, *GFI* = 0.99, *CFI* = 1.00, *RMR* = 0.01, *RMSEA* = 0.00, satisfactory internal consistency (α = 0.90), and an average inter-item correlation of *r* = 0.70. Thus, the 4-item measure was used in the study (Appendix B).

#### Prevalence of flow experience at work

To limit the time it took to participate in the study and simultaneously capture a global measure of flow experience (rather than focusing on its dimensions), the prevalence with which work-related flow is experienced was measured using an abridged version of one of the oldest flow measures that exist, the Flow Questionnaire (Csikszentmihalyi and Csikszentmihalyi, 1988). Participants were asked, “In your current job, how often do you experience flow while doing work?” Responses were reported using a 5-point (1 = *never*; 2 = *once a year or more but not every month*; 3 = *once a month or more but not every week*; 4 = *once a week or more but not every day*; 5 = *every day*) Likert scale.

#### Level of social interaction characterizing work

The level of social interaction characterizing participants' jobs was calculated by summing 38 element ratings across 4 O^*^NET categories-subcategories for the occupational entries they provided: Work Activities—Interacting with Others, Work Context—Interpersonal Relationships, Skills—Social Skills, and Interests—Social. O^*^NET is an online database developed under the sponsorship of the U.S. Department of Labor/Employment and Training Administration that contains occupation-specific ratings of job characteristics for nearly 1,000 unique occupations. These ratings are standardized scores that range from 0 to 100 and indicate the degree to which a particular descriptor is required to perform an occupation (O^*^NET Online, 2018).

#### RIASEC vocational interests

The extent to which work fulfills artistic and conventional vocational interests was also measured using archival data from O^*^NET, which rates occupations using Holland's (1985) R-I-A-S-E-C Interest Structure (Rounds et al., 1999). The RIASEC ratings on O^*^NET were developed and validated using both judgmental and empirical methods, with the degree to which work environments are characterized by the dimensions being reported on a 7-point (1 = not at all characteristic, 7 = extremely characteristic) scale (Rounds et al., 1999). *Artistic* occupations commonly involve working with forms, designs, and patterns. They frequently require self-expression, and the work can be done without following a clear set of rules. *Conventional* occupations, on the other hand, commonly involve following set procedures and routines. They often include working with data and details more than with ideas, and there is usually a clear line of authority to follow (O^*^NET Online, 2018).

### Study 1 results

Descriptive statistics and bivariate correlations are presented in Table 1. Consistent with the expectation that workers vary in the extent to which they believe work-related flow is useful for the jobs they perform, noteworthy variance was observed in flow metacognition; with 11.2% of the sample not agreeing (*M* = 1.00–3.00), 63.6% moderately agreeing (*M* = 3.01–4.50), and 25.2% strongly agreeing (*M* = 4.51–5.00) that flow benefits job performance. *Hypothesis 1* proposed that metacognitive beliefs about the utility of work-related flow for job performance would positively predict the frequency with which workers experience flow on the job. The results of a simple linear regression conducted using JAMOVI (The JAMOVI Project, 2020) provide initial support for this hypothesis, *R* = 0.39, *R*^2^ = 0.15, *F*_(1, 391)_ = 68.7, *p* < 0.001, with an unstandardized coefficient for flow metacognition predicting prevalence of flow of *b* = 0.57, *p* < 0.001, 95% CI (0.44, 0.71). Additionally, results of a multiple linear regression conducted using JAMOVI (The JAMOVI Project, 2020) provide initial support [*R* = 0.21, *R*^2^ = 0.04, *F*_(3, 388)_ = 5.78, *p* < 0.001] for levels of social interaction [*b* = −0.004, *p* = 0.007, 95% CI (−0.01, −9.88e-4)] and the extent to which work fulfills artistic (*b* = 0.005, *p* = 0.012, 95% CI (0.001, 0.008)] and conventional vocational interests [*b* = 0.005, *p* = 0.016, 95% CI (0.001, 0.009)] predicting flow metacognition.

**Table 1:** Descriptive statistics and bivariate correlations for study 1.

	**M (SD)**	**95% CI**	**1**	**2**
Flow metacognition	4.04 (0.82)	(3.96, 4.12)	–	–
Prevalence of flow experience	3.58 (1.19)	(3.46, 3.70)	0.37^*^	–

* *p* < 0.05.

Scores transformed using log10 transformation to increase normality.

### Study 1 discussion and rationale for subsequent studies

Study 1 advances the understanding of metacognitive beliefs about the utility of work-related flow for job performance and its nomological network. CFA results provided initial validation for the psychometric validity of the measure, while descriptive statistics suggest evidence for the variance in metacognitive beliefs among workers regarding how useful flow is for performance in their jobs. Study 1 also provided initial insights into factors that may influence these beliefs (i.e., levels of social interaction and the fulfillment of artistic and conventional vocational interests). Workers meaningfully varied in terms of their utility perceptions, with this variance significantly related to levels of social interaction characterizing their jobs and their jobs' fulfillment of artistic and conventional vocational interests.

Metacognitive beliefs about the utility of work-related flow for job performance were also found to significantly relate to the frequency with which workers reported experiencing flow on the job. Subsequently, these findings provide initial support for metacognitive beliefs about the utility of work-related flow for job performance being an important antecedent of the state, provided insight into the theory of the construct, and provided support for the psychometric validity of a new measure of usefulness beliefs about the utility of work-related flow for job performance. Nevertheless, this research was cross-sectional, utilized a single-item measure of flow, and did not include the other elements of cognitive control that are hypothesized to influence flow at work (i.e., grit and mindfulness) or its associated outcomes (i.e., engagement, performance, and burnout). As such, study 2 was conducted to provide an initial test of the CCMWF by utilizing a time-lag approach, a more robust measure of flow, and included the antecedents of flow metacognition, mindfulness, and grit.

### Study 2 participants

A 2-week time-lag study was conducted with an initial sample of *n* = 295 workers in the United States who work at least 25 h per week and were 18 years of age or older and were recruited through Amazon Mechanical Turk (MTurk). Participants were removed from the data set if they indicated that they did not have a full-time job besides MTurk, completed the study exceptionally fast, selected incorrect answers to embedded attention checks, or if it was determined that their responses were completed carelessly. The final sample for time 1 included *n* = 286 participants with representation from every industry recorded by the U.S. Bureau of Labor Statistics (2019). Two weeks later, a follow-up survey was distributed which resulted in *n* = 207 participants with complete data (Table 2).

**Table 2:** Study 2 sample demographics.

	**Time 1 statistics**	**Time 2 statistics**
N	287	207
Males	38.6%	36.1%
Caucasian	69.5%	71.6%
Mean age	35.8	37.6
Years of work experience	22.3	16.4
Years of job tenure in current positions	6.3	7

### Design and procedure

In accordance with the recommendations of Podsakoff et al. (2003) for mitigating common method variance, the study utilized a 2-week time-lagged design in which the predictors and criterion were recorded separately, counter-balanced, and in which scales of different Likert scale lengths were utilized. Participants were given an initial survey measuring flow, grit, trait mindfulness, flow metacognition, and demographic data. After 2 weeks, participants completed a second survey measuring performance, engagement, and burnout.

#### Study 2 measures

Unless otherwise indicated, participants responded to all survey items using a 5-point Likert scale (1 = *strongly disagree* to 5 = *strongly agree*).

##### Psychological grit

Psychological grit was measured using 5 items adapted from the Grit-S scale developed by (Duckworth and Quinn, 2009; α = 0.755). An example item is, “Setbacks don't discourage me.”

##### Flow metacognition

Three items retained from the Flow Metacognition scale developed in study 1 were used to assess beliefs about the usefulness of work-related flow for job performance (α = 0.868). An example item includes, “Regularly experiencing flow would enhance your overall job performance.”

##### Flow prevalence

Six items from the work domain dimension of the English translation of the Swedish Flow Proneness Questionnaire (Ullén et al., 2012; α = 0.777) were used to assess the prevalence of flow. A sample item includes: “When you do something at work, how often does it happen that it feels as if your ability to perform what you do completely matches how difficult it is?” (1 = *never*; 5 = *every day*).

##### Trait mindfulness

Trait mindfulness (one's propensity for experiencing mindfulness) was measured using 12 items from Brown and Ryan's (2003) Mindfulness Attention and Awareness scale (α = 0.929). An example item includes, “I find it difficult to stay focused on what's happening in the present” (reverse coded).

##### Burnout

Burnout was measured utilizing a seven-item adaptation of the Shirom–Melamed Burnout Measure (SMBM; Melamed et al., 1992; α = 0.945). An example item is, “In the past two weeks at work, how often did it happen that you felt you were not capable of investing emotionally in coworkers and customers?” (1 = *never or almost never*; 7 = *always or almost always*).

##### Engagement

Engagement was measured using 10 items from Rich et al.'s (2010) Job Engagement scale (α = 0.958). An example item is, “I work with intensity on my job” (1 = strongly disagree; 7 = strongly agree).

##### Job performance

Job performance was measured utilizing three items of Griffin et al.'s (2007) Work Performance scale (α = 0.875). An example item included, “In the past two weeks at work, how often did it happen that you carried out the parts of your job well” (1 = never or almost never; 7 = always or almost always).

#### Study 2 results

A series of CFA was conducted using JASP version 0.13.1 (JASP Team, 2020) to ensure an acceptable measurement model. Initially, all 58 items from the seven scales were included in the analyses. However, given that the CCMWF includes constructs that are strongly theoretically related, many of the items between the scales are very similar (i.e., related to attention) and have shared variance. As such, initial fit measures for the measurement model were unsatisfactory (*RMSEA* > 0.08, *CFI* < 0.9, and *TLI* < 0.9). Therefore, items were removed from the measurement model based on the largest modification indices, ensuring that all scales had at least three items and that reliability coefficients for each scale maintained acceptable levels. The resulting measurement model included 45 items and demonstrated close fit [$\chi_{\left(924\right)}^{2}$ = 1,420, *p* < 0.001, *CFI* = 0.922, *TLI* = 0.916, *RMSEA* = 0.051; Browne and Cudeck, 1992]. The information presented in the measures section reflects these modifications, and all reliability coefficients exceeded the lower limit for Cronbach's alpha of 0.70 (Hair et al., 2009). Additionally, a correlation matrix (and descriptive statistics; Tables 3, 4) was calculated to explore initial correlations between the variables included in the model and the overall study.

**Table 3:** Study 2 descriptive statistics.

	**Flow proneness**	**Flow metacognition**	**Grit**	**Trait mindfulness**	**Burnout**	**Engagement**	**Performance**
*N*	286	286	286	286	207	207	207
Missing	0	0	0	0	79	79	79
Mean	3.54	3.79	3.56	3.25	3.20	5.39	4.73
Median	3.57	4.00	3.50	3.20	3.20	5.61	4.67
Standard deviation	0.586	0.875	0.680	0.934	1.38	1.08	1.01
Variance	0.343	0.766	0.462	0.873	1.90	1.16	1.01
Range	3.00	4.00	3.13	4.00	5.34	5.72	4.67
Minimum	2.00	1.00	1.88	1.00	1.00	1.28	2.33
Maximum	5.00	5.00	5.00	5.00	6.34	7.00	7.00

**Table 4:** Study 2 bivariate correlations.

	**Trait mindfulness**	**Flow proneness**	**Flow metacognition**	**Grit**	**Burnout**	**Engagement**	**Performance**
Trait mindfulness	—						
Flow proneness	0.13^*^	—					
Flow metacognition	−0.14^*^	0.15^**^	—				
Grit	0.69^***^	0.18^**^	−0.16^**^	—			
Burnout	−0.55^***^	−0.30^***^	0.07	−0.55^***^	—		
Engagement	0.25^***^	0.52^***^	0.07	0.21^**^	−0.41^***^	—	
Performance	0.30^***^	0.49^***^	0.11	0.20^**^	−0.42^***^	0.63^***^	—

* *p* < 0.05,

** *p* < 0.01,

*** *p* < 0.001.

As illustrated in Figure 1, Structural Equation Modeling (SEM) with a Diagonally Weighted Least Squares estimator (Mîndrilá, 2010; Li, 2016) was utilized to test *Hypothesis 1* (Metacognitive beliefs about the utility of work-related flow for job performance will positively predict the frequency with which workers experience flow on the job), *Hypothesis 2* (Mindfulness will positively predict the frequency with which workers experience flow on the job), *Hypothesis 3* (Psychological grit will positively predict the frequency with which workers report experiencing flow on the job), and the extent to which the observed data fit the overall CCMWF proposed in this study. Mindfulness, grit, and flow metacognition were each entered into the model as predictors of flow, burnout, engagement, and performance. Flow was also entered as a predictor of burnout, engagement, and performance.

The criteria used to assess the sufficient fit of the model included *RMSEA* < 0.08, *CFI* > 0.9, and *TLI* > 0.9. Additionally, hypotheses were considered supported if *p* < 0.05 for the proposed relationship within the model. For example, if trait mindfulness predicted flow at *p* < 0.05, this would indicate support for Hypothesis 2 (Mindfulness will positively predict the frequency with which workers experience flow on the job). The tested model demonstrated good fit, $\chi_{\left(924\right)}^{2}$ = 656.21, *CFI* = 1.00, *TLI* = 1.02, *RMSEA* = 0.00, *SRMR* = 0.06, providing support for the CCMWF. Results of the SEM suggest that, consistent with *Hypothesis 1*, the flow metacognition positively predicted flow, *b* = 0.05, *SE* = 0.02, *p* = 0.001, 95% CI (0.02, 0.08), *β* = 0.13. *Hypothesis 2* was also supported, in that trait mindfulness positively predicted flow, *b* = 0.09, *SE* = 0.03, *p* = 0.007, 95% CI (0.03, 0.16), *β* = 0.22. Additionally, *Hypothesis 3* was supported, in that grit positively predicted flow, *b* = 0.11, *SE* = 0.04, *p* = 0.013, 95% CI (0.02, 0.20), *β* = 0.24. These results suggest that those higher in trait mindfulness are likely to experience flow more frequently than those lower in trait mindfulness. As such, *hypotheses 1, 2*, and 3 were supported.

In addition to the analyses testing the *hypotheses*, indirect effects were calculated in which flow mediates the relationship between each of the independent variables and each of the outcome variables (*hypotheses* 4abc-6abc) using JASP version 0.13.1 (JASP Team, 2020). Results suggest that flow mediated the relationship between trait mindfulness and performance [*b* = 0.18, *SE* = 0.07, *p* = 0.004, 95% CI (0.06, 0.31), *β* = 0.13], burnout [*b* = −0.05, *SE* = 0.02, *p* = 0.04, 95% CI (−0.09, −0.002), *β* = −0.03], and engagement [*b* = 0.16, *SE* = 0.06, *p* = 0.005, 95% CI (0.05, 0.27), *β* = 0.13]. Additionally, flow meditated the relationship between flow metacognition and performance [*b* = 0.10, *SE* = 0.03, *p* = 0.002, 95% CI (0.04, 0.16), *β* = 0.08], burnout [*b* = −0.02, *SE* = 0.01, *p* = 0.008, 95% CI (−0.04, −0.01), *β* = −0.02], and engagement [*b* = 0.09, *SE* = 0.03, *p* = 0.002, 95% CI (0.03, 0.14), *β* = 0.08]. Finally, flow meditated the relationship between grit and performance [*b* = 0.22, *SE* = 0.10, *p* = 0.02, 95% CI (0.03, 0.41), *β* = 0.14], burnout [*b* = −0.05, *SE* = 0.02, *p* = 0.009, 95% CI (−0.10, −0.01), *β* = −0.03], and engagement [*b* = 0.19, *SE* = 0.08, *p* = 0.017, 95% CI (0.03, 0.35), *β* = 0.14]. As such, support was found for *hypotheses 4abc*−*6abc*.

#### Study 2 discussion and rationale for study 3

Grit, flow metacognition, and trait mindfulness were all found to mediate the relationship between flow and engagement, burnout, and performance at work. These results contribute to the theory of the construct of flow at work by providing further support for flow metacognition as an antecedent of flow at work and integrating these initial findings into a broader model. Through the lens of COR theory (Hobfoll, 1989), support was found for mindfulness and grit as previously untested antecedents of flow at work relate to the ability to focus cognitive resources toward flow. This research also extends findings related to flow predicting the outcomes of engagement, burnout, and performance (Bakker et al., 2011; Lavigne et al., 2012; De Fraga and Moneta, 2016) by finding support for these relationships in a population of workers from a wide array of industries and integrating these variables into a single supported model of optimal performance at work. However, study 2 focused on trait-level measures, and further research which measures flow and mindfulness at the state level to explore these relationships at both within and between persons is needed. While measures of trait mindfulness and flow proneness provide important insights into how frequently individuals believe that they experience these states, state-level measures provide more accurate accounts of how frequently these states are experienced, fluctuations in these states throughout the day, and enable the examination of within-person variance. As such, the purpose of study 3 is to provide further validation for the CCMWF by utilizing an experience-sampling design that measures mindfulness and flow at the state level.

#### Study 3 participants

A sample of *n* = 173 workers in the United States who work at least 25 h per week and were 18 years of age or older was recruited through Amazon Mechanical Turk (MTurk). Qualifying participants who answered attention checks correctly and who did not miss more than one of the four surveys during the day were compensated either $2 or 2.50^1^ for their participation and were entered into a raffle for a $50 Amazon gift card. Those who do not meet these criteria were not compensated for incomplete data. This resulted in a sample of *n* = 162, where 56% of participants were male and 81.1% were Caucasian. Participants worked mostly in the for-profit sector, across a multitude of industries, and ranged from ages 20–68 years. While these demographics are roughly comparable to the US population, there was an overrepresentation of white participants (81 vs. 63%), and an underrepresentation of government workers (7 vs. 15%; Descriptive statistics shown in Table 5 with comparisons for Sex, Ethnicity, and Type of Organization to the overall U.S. population according to the 2016 US Bureau of Labor Statistics report). Additionally, over 130 unique job titles were reported in the sample, with diverse positions ranging from a high degree of managerial responsibilities (i.e., Director) to lower levels of managerial responsibilities (i.e., Associate), “white collar” jobs (i.e., System Administrator), and “blue collar” jobs (i.e., Iron Worker).

**Table 5:** Study 3 descriptive statistics.

	**Position tenure**	**Organization tenure**	**Workforce tenure**	**Age**
N	159	159	159	159
Missing	3	3	3	3
Mean	6.06	7.04	16.7	36.8
Median	4.00	6.00	15	35
Minimum	0.00	0.00	2	20
Maximum	39.0	29.0	54	68
**Levels**	**Study sample counts**	**% of study sample**	**Overall % US population (U.S. Bureau of Labor Statistics**, **2016****)**
**Frequencies for study 3**			
**Frequencies of organization type in study 3**			
For profit	132	83.0%	76.27%
Non-profit	16	10.1%	8.67%
Government	11	6.9%	15.06%
**Levels**	**Counts**	**% of total**
**Study 3: frequencies of industry in study 3**		
Accommodation and food services	8	5.0%
Construction	6	3.8%
Educational services	9	5.7%
Finance and insurance	14	8.8%
Government	3	1.9%
Health care and social assistance	17	10.7%
Information technology	22	13.8%
Manufacturing	12	7.5%
Other services (except public administration)	8	5.0%
Professional, scientific, and technical services	24	15.1%
Real estate and rental and leasing	2	1.3%
Retail trade	15	9.4%
Transportation or warehousing	2	1.3%
Utilities	2	1.3%
Wholesale Trade	1	0.6%
Other	2	1.3%
Administrative and support services	3	1.9%
Agriculture, forestry, fishing, and hunting	1	0.6%
Arts, entertainment, and recreation	8	5.0%
**Levels**	**Counts**	**% of study sample**	**Overall % US population (U.S. Bureau of Labor Statistics**, **2016****)**
**Study 3: frequencies of sex in study 3**			
Male	89	56.0%	53.2%
Female	70	44.0%	46.8%
**Study 3: frequencies of race in study 3**			
Hispanic or Latino	8	5.0%	16.8%
White (Not Hispanic or Latino)	129	81.1%	63.4%
Black or African American	14	8.8%	12.3%
Asian (Not Hispanic or Latino)	5	3.1%	6%
Other [The “all other groups” category includes (1) those classified as of being of multiple racial origin and (2) the race categories of (2a) American Indian and Alaska Native and (2b) Native Hawaiian and Other Pacific Islanders.]	1	1.9%	3.3%

#### Study 3 design and procedure

A 1-day experience sampling method (ESM) design (Csikszentmihalyi and Larson, 1987) was utilized to test the relationships proposed by the CCMWF. An initial survey was administered before starting the ESM to record grit, flow metacognition, and demographic data, flow and state mindfulness were measured during the day and engagement, performance, and burnout were measured at the end of the day. Instructions for participants to download and enter a study code in the ExpiWell (Tay, 2015) smartphone app to participate in the ESM portion of the study were disseminated at the end of the initial survey.

Participants were alerted four times on the day following time 0, over an 8-h period (to sample mindfulness and flow throughout a typical workday), with at least 1 h between alerts. Each time participants were sent an alert, they were asked to complete a short, self-report survey asking whether they were at home, work, or other (with the ability to specify the location through open-ended text), as well as state measures of flow and mindfulness. The location information was utilized to remove data that were unrelated to work to ensure that work-related flow remains the focus of the analyses. Participants were given a 2-h window to take each survey before it expired and received a reminder notification if they did not complete the survey after the first hour. At the end of the day, participants were given a final survey asking them to report measures of engagement, performance, and burnout. Following the recommendations of Podsakoff et al. (2003), predictor (i.e., grit, flow metacognition, mindfulness, and flow) and criterion variables (burnout, engagement, and performance) were measured at different points in time, and counterbalanced to minimize the effects of common method bias.

#### Study 3 measures

Unless otherwise indicated, participants responded to all survey items using a 5-point Likert scale (1 = *strongly disagree* to 5 = *strongly agree*).

#### Psychological grit

Psychological grit was measured using four items adapted from the Grit-S scaled developed by Duckworth and Quinn (2009; α = 0.77). An example item is, “Setbacks don't discourage me.”

#### Flow metacognition

Three items from the Flow Metacognition scale developed in study 1 (and utilized in study 2) were used to assess beliefs about the usefulness of work-related flow for job performance (α = 0.92). An example item includes “Regularly experiencing flow would enhance your overall job performance.”

#### Burnout

Burnout was measured utilizing an eight-item adaptation of the Shirom–Melamed Burnout Measure (α = 0.91; SMBM; Melamed et al., 1992). An example item is, “I have no energy for going to work in the morning.”

#### Engagement

Engagement was measured using five items adapted from the Job Engagement scale (α = 0.79) developed by Rich et al. (2010). An example item is “I exert my full effort to my job”.

#### Flow prevalence

Flow was measured using seven items from the Short Flow State scale (Jackson et al., 2008; α _between_ = 0.90, α_within_ = 0.63). An example item includes: “I had a feeling of total control over what I was doing.”

#### State mindfulness

State mindfulness was measured using Brown and Ryan's (2003) five-item state version of the Mindfulness Attention and Awareness scale (α_between_ = 0.91, α_within_ = 0.66). An example item includes, “I found myself preoccupied with the future or the past” (reverse coded).

#### Subjective performance

Subjective performance was measured utilizing four items adapted from Griffin et al.'s (2007) Work Performance scale (α = 0.77). An example item utilized is, “When thinking about your work today, how often did it happen that you carried out the parts of your job well” (1 = never or almost never; 5 = always or almost always).

### Study 3 results

A series of multilevel confirmatory factor analyses (MCFA) was conducted using MPlus version 8.4 (Muthén and Muthén, 2019) with the maximum likelihood estimator to ensure an acceptable measurement model. However, the model could not converge, likely due to the sheer number of items and relatively low sample size. Additionally, given that the CCMWF includes constructs that are strongly theoretically related, many of the items between the scales are very similar (i.e., related to attention) and have shared variance. Therefore, items were removed from the measurement model based on the largest modification indices, ensuring that all scales had at least three items and that reliability coefficients for each scale maintained acceptable levels. The resulting measurement model demonstrated close fit [$\chi_{\left(696\right)}^{2}$ = 3,878.65, *p* < 0.001, *CFI* = 0.922, *TLI* = 0.913, *RMSEA* = 0.03; Browne and Cudeck, 1992]. The information presented in the measures section reflects these modifications. Additionally, all between-person reliability coefficients exceeded the lower limit for Cronbach's alpha of 0.70 (Hair et al., 2009). While the within-person Cronbach's alphas for state mindfulness and state flow fell below this threshold, Nezlek (2017) suggests that the rules for within-level reliability should be relaxed because within-level reliability is frequently lower than typical between-level reliability. Additionally, Shrout's (1998) classification system suggests that an alpha coefficient from 0.61 to 0.80 can be considered to have moderate reliability. Therefore, all measures were shown to have acceptable reliability coefficients. Additionally, a correlation matrix (and descriptive statistics; Tables 6, 7) was calculated to explore initial correlations between the variables included in the model and the overall study.

**Table 6:** Descriptive statistics and correlation matrix for the variables of interest in study 3.

	**State flow**	**Mindfulness**	**Burnout**	**Engagement**	**Performance**	**Grit**	**Flow metacognition**
*N*	448	448	155	155	155	159	159
Mean	4.11	4.07	2.00	4.37	4.54	3.33	4.16
Median	4.14	4.20	1.75	4.40	4.75	3.50	4.00
Standard deviation	0.61	0.84	0.96	0.57	0.54	0.97	0.75
Minimum	1.71	1.40	1.00	2.60	2.50	1.00	1.33
Maximum	5.00	5.00	4.63	5.00	5.00	5.00	5.00

**Table 7:** Study 3 correlation matrix.

	**State flow**	**State mindfulness**	**Performance**	**Flow metacognition**	**Grit**	**Burnout**	**Engagement**	**Age**	**Sex**
State flow	—								
State mindfulness	0.68^***^ (0.41^***^)	—							
Performance	0.50^***^	0.33^***^	—						
Flow metacognition	0.17^*^	−0.01	0.20^**^	—					
Grit	0.40^***^	0.46^***^	0.13	−0.14	—				
Burnout	−0.54^***^	−0.56^***^	−0.26^***^	0.02	−0.48^***^	—			
Engagement	0.55^***^	0.45^***^	0.47^***^	0.17^*^	0.29^***^	−0.41^***^	—		
Age	0.17^*^	0.23^***^	0.08	−0.12	0.11	−0.15	0.14^**^	—	
Sex	0.14	0.16^*^	0.07	−0.01	−0.08	0.09	0.04	0.29^***^	—
Race	0.06	0.11	−0.03	0.03	0.13	−0.01	0.01	−0.16	−0.09

* *p* < 0.05,

** *p* < 0.01,

*** *p* < 0.001; Relationships in parentheses are at the within-level. All other relationships are at the between-level.

### Study 3 hypothesis testing

As illustrated in Figure 1, a multilevel structural equation model (MSEM; Kline, 2015) was utilized to test *Hypothesis 1* (Metacognitive beliefs about the utility of work-related flow for job performance will positively predict the frequency with which workers experience flow on the job), *Hypothesis 2* (Mindfulness will positively predict the frequency with which workers experience flow on the job), *Hypothesis 3* (Psychological grit will positively predict the frequency with which workers report experiencing flow on the job), and the extent to which the observed data fit the overall CCMWF proposed in this study using MPlus version 8.4 (Muthén and Muthén, 2019). Mindfulness, grit, and flow metacognition were each entered into the model as predictors of flow, burnout, engagement, and performance. Flow was also entered as a predictor of burnout, engagement, and performance. To control for within-individual variance, the flow and mindfulness variables were both clustered by Participant ID number.

**Figure 2:**
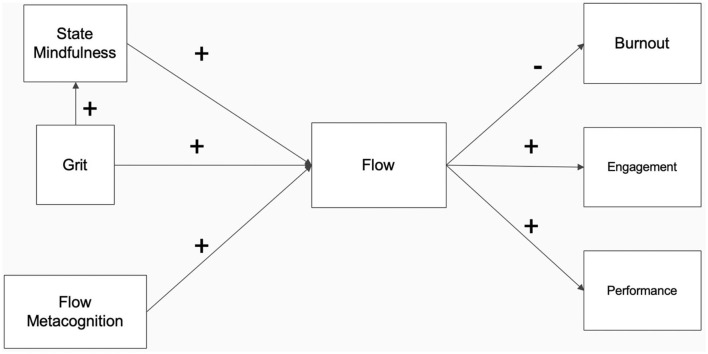
Alternate Cognitive Control Model of Work-related Flow with grit predicting state mindfulness supported in study 3. Direct effects of mindfulness, grit, and flow metacognition on burnout, engagement, and performance were removed from the figure for parsimony but were included in analyses. All displayed variables represent latent constructs.

The criteria used to assess the sufficient fit of the model included *RMSEA* < 0.08, *CFI* > 0.9, and *TLI* > 0.9. Additionally, hypotheses were considered supported if *p* < 0.05 for the proposed relationship within the model. For example, if state mindfulness predicts flow at *p* < 0.05, this would indicate support for *Hypothesis 2*. The analysis utilized a Full Information Maximum Likelihood Estimation (FIML), which allows for missing data to be ignored, does not impute data, and is more efficient and less biased than other methods used for dealing with missing data (Enders and Bandalos, 2001). However, the results of the MSEM indicated a poor model fit (*RMSEA* = 0.163, *CFI* = 0.888, *TLI* = 0.175); therefore, a new model was tested based on modification indices, which indicated that a single path be added in which grit predicts state mindfulness (Figure 2).

The alternate CCMWF with grit predicting state mindfulness demonstrated acceptable fit, *CFI* = 0.996, *TLI* = 0.952, *RMSEA* = 0.04, providing support for the model. The intraclass correlation (ICC) for the within-person variables of state mindfulness and flow were considerable (0.536 and 0.439, respectively) (Table 8), suggesting that the within-person variance of the daily variables ranged from 44 to 54%, justifying the use of multilevel analysis for the current study (Tse et al., 2020). Results of the MSEM (Table 9) suggest that consistent with *Hypothesis 1*, flow metacognition positively predicted flow, *b* = 0.13, *SE* = 0.03, *p* < 0.001, 95% CI (0.07, 0.13), *β* = 0.24. *Hypothesis 2* was also supported both the within- and between-person levels, in that state mindfulness positively predicted flow at both the within-, *b* = 0.33, *SE* = 0.06, *p* < 0.001, 95% CI (0.23, 0.43), *β* = 0.41, and between-person levels, *b* = 0.46, *SE* = 0.08, *p* < 0.001, 95% CI (0.33, 0.59), *β* = 0.69. Additionally, *Hypothesis 3* was supported (as an indirect relationship rather than a direct relationship as initially proposed), in that grit positively predicted flow indirectly through state mindfulness, *b* = 0.16, *SE* = 0.03, *p* < 0.001, 95% CI (0.11, 0.21), *β* = 0.38. However, the direct relationship between grit and flow was non-significant, *p* = 0.06. These results suggest that those who experience more state mindfulness are likely to experience flow more frequently than those with lower state mindfulness, and, that when individuals experience state mindfulness, they are more likely to also experience more flow within themselves throughout that day. As such, *hypotheses 1, 2*, and *3* were supported.

**Table 8:** Intraclass correlations (ICC).

	**State flow**	**State mindfulness**
ICC (1)	0.44	0.54

**Table 9:** Results of Alternate Cognitive Control Model of Work-related Flow with grit predicting state mindfulness.

	**Performance estimates (SE)**	**Engagement estimates (SE)**	**Burnout estimates (SE)**	**State flow estimates (SE)**	**State mindfulness estimates (SE)**
Intercept	0.76 (0.55)	0.16 (0.48)	7.53 (0.80)^***^	1.50 (0.27)^***^	2.91 (0.18)^***^
State mindfulness (within-person)	–	–	–	0.33 (0.06)^***^	–
State mindfulness (between-person)	−0.13 (0.16)	0.05 (0.17)	−0.40 (0.28)	0.46 (0.08)^***^	–
Grit	−0.12 (0.05)^**^	−0.02 (0.06)	−0.15 (0.11)	0.07 (0.04)	0.35 (0.05)^***^
Flow metacognition	0.06 (0.07)	−0.02 (0.06)	0.09 (0.10)	0.13 (0.03)^***^	–
State flow	1.08 (0.28)^***^	1.11 (0.25)^***^	−0.93 (0.45)^*^	–	–
Total indirect effect of state mindfulness (between-person) through state flow	0.49 (0.16)^**^	0.50 (0.15)^**^	−0.42 (0.20)^*^	–	–
Total indirect effect of grit through state flow	0.07 (0.04)	0.07 (0.04)	−0.06 (0.05)	–	–
Total indirect effect of flow metacognition through state flow	0.14 (0.05)^**^	0.14 (0.05)^**^	−0.12 (0.06)	–	–
Total indirect effect of grit through state mindfulness (between)	–	–	–	0.16 (0.03)^***^	–
*R^2^*	0.43	0.50	0.49	0.67_between_ 0.17_within_	0.30_between_

* *p* < 0.05,

** *p* < 0.01,

*** *p* < 0.001; All estimates are unstandardized.

Indirect effects were calculated to test hypotheses 4abc−6abc in which flow mediates the relationship between each of the independent variables and each of the outcome variables using MPlus version 8.4 (Muthén and Muthén, 2019). Results suggest that flow mediates the relationship between state mindfulness and performance [*b* = 0.49, *SE* = 0.16, *p* = 0.002, 95% CI (0.23, 0.76), *β* = 0.69], burnout [*b* = −0.42, *SE* = 0.20, *p* = 0.04, 95% CI (−0.75, −0.09), *β* = −0.27], and engagement [*b* = 0.50, *SE* =0.05, *p* < 0.001, 95% CI (0.26, 0.75), *β* = 0.53]. Additionally, flow meditated the relationship between flow metacognition and performance [*b* = 0.14, *SE* = 0.05, *p* = 0.006, 95% CI (0.06, 0.22), *β* = 0.19] and flow metacognition and engagement [*b* = 0.14, *SE* = 0.05, *p* = 0.002, 95% CI (0.07, 0.22), *β* = 0.18]. Flow did not mediate the relationship between grit and any of the outcomes (*p* > 0.05) and is marginally significant for the relationship between flow metacognition and burnout [*b* = −0.12, *SE* = 0.06, *p* = 0.055, 95% CI (−0.22, −0.02), *β* = −0.09]. As such, support was found for *hypotheses* 4ac−6c, but not *hypotheses* 4b, 5b, 6c (i.e., flow mediating the relationship between grit and the outcome variables), or 6a (flow mediating the relationship between flow metacognition and burnout).

### Study 3 preliminary discussion

Study 3 addresses multiple limitations of studies 1 and 2. Namely, the study utilized an experience sampling methodology and a state-based measure of mindfulness and flow to analyze the CCMWF at the between and within levels. The study provided further support for all the proposed relationships between the antecedents of flow at work, its outcomes, and a slightly revised version of the CCMWF (Figure 2), except for flow mediating the relationship between grit and the outcome variables, or flow mediating the relationship between flow metacognition and burnout.

## General discussion

The current research aimed to provide empirical support for the “Cognitive Control Model of Work-related Flow.” While previous research has proposed models which include job characteristics, job resources, job demands, and personal resources as antecedents of flow at work (Demerouti and Mäkikangas, 2017), the current model focused on how workers might actively pursue flow at work through their own volition, and how they can develop the competencies necessary for effectively pursuing flow on the job. Therefore, the current research expanded the nomological network of flow at work by answering distal calls for research exploring the relationship between flow and grit (Duckworth, 2016; Smith et al., 2020), and the relationship between flow and mindfulness at work (Dust, 2015; Ceja and Navarro, 2017; Marty-Dugas et al., 2020; Weintraub and Dust, 2020) calls to better understand the relationship between flow and personal resources and provide a foundation for interventions aimed at increasing flow at work (Bakker and van Woerkom, 2017; Demerouti and Mäkikangas, 2017). Additionally, this research shed further light on the relationship between flow and metacognitive beliefs (Wilson and Moneta, 2016). While these calls for research have come from a plethora of authors across the globe, they are all related in that they focus on different aspects of one's motivation and ability to exercise cognitive control toward experiencing flow at work.

Therefore, the current research aimed to integrate these constructs over the course of three studies into a cohesive CCMWF which includes antecedents that explain an individual's motivation for experiencing flow (flow metacognition), the ability to allocate resources toward focusing on and persevering in the pursuit of flow (grit), the ability to purposefully exert cognitive control toward experiencing flow at work (mindfulness), and subsequently, that flow will predict the outcomes of engagement, burnout, and performance. By examining these relationships as part of an integrated model, not only can the direct relationships of these understudied antecedents be considered, but these relationships can be more effectively examined more broadly and holistically. This holistic point of view provides the foundation for exploring the antecedents which have the largest effect on flow at work in the context of a wider nomological network, whether these relationships have downstream effects on the crucial work outcomes included in the model (i.e., indirect effects from antecedents, to flow, to outcomes), and lays the foundation for identifying and validating interventions which are likely to be most effective for influencing flow and the outcomes of interest for researchers and practitioners alike.

While the proposed model was supported at the trait level in study 2, study 3 required minimally revising the model (to include a single path from grit to state mindfulness) in a way that still aligns with the tenets of the CCMWF. Results of study 3 indicated a model, which not only demonstrated good fit (Browne and Cudeck, 1992) but also explained between 17 and 67% of the variance in the constructs of interest (Table 9), indicating medium to large effect sizes (Cohen, 1988). These results suggest that not only is the revised model an appropriate lens for researchers and practitioners to view these relationships but the model also explains a large (five out of the six *R*^2^values are equal to or >0.25) amount of the variance in the variables of interest. This is especially true for the focal variable of flow, as the revised model explains 67% of the between-person variance and 17% of the within-person variance. Overall, these results suggest that, together, variables related to one's motivation and ability to direct cognitive control toward experiencing flow explain a great deal of the variance in the prevalence of state flow in the workplace. While these findings are encouraging for initial empirical support of the overall revised CCMWF, by digging a bit deeper into the individual relationships and viewing them in the context of the overall model, these results provide greater insights into the mechanisms behind these relationships, and where researchers and practitioners should focus their future efforts to help individuals and organizations thrive.

First, the results provide further support for the notion that flow positively predicts performance and engagement, while negatively predicting burnout. While these results have been demonstrated in previous research, the replication of these findings provides further support for these relationships, even when put into the broader context of a model which includes other constructs such as grit and mindfulness, which have also been associated with these outcomes (Grégoire and Lachance, 2015; Duckworth, 2016; Coo and Salanova, 2018; Hafenbrack and Vohs, 2018). This indicates that flow is a key driving force behind these crucial outcomes in the model for individuals and organizations. As such, practitioners would be wise to consider how they can help promote flow in the field to drive these crucial work outcomes. Furthermore, flow should be included as a variable in research examining mindfulness or grit, and their relationships with performance, engagement, or burnout to ensure a more accurate examination of these relationships.

With regard to the proposed antecedents of flow, support was found for the nascent construct of flow metacognition and for the Flow Metacognition scale developed in study 1 as a means by which to assess beliefs about the usefulness of work-related flow for job performance. Support was also found for flow metacognition, grit, and mindfulness (both within- and between levels) as antecedents of flow in both studies 1 and 2. Knight and Waples (2017) suggest that “in the context of work, motivation theories are…focused on understanding factors that determine why workers engage (or fail to engage) in specific work-related tasks, the effort (typically conceptualized as time and resources expended) applied to those tasks, and the degree to which they maintain engagement in the tasks across time” (p. 142). In accordance with this assertion, and in line with the COR Theory (Hobfoll, 1989), and popular theories of motivation (e.g., Vroom, 1964), behavior (Ajzen, 1988), and decision-making (e.g., Tversky and Kahneman, 1992), these results provide support for the CCMWF in that the prevalence of state flow at work differed between (and within) employees, depending on their motivation for experiencing flow (flow metacognition), their ability to allocate resources toward focusing on, and resiliently pursuing flow (grit), and their ability to purposefully exert cognitive control toward experiencing flow at work (mindfulness).

However, it must be noted that while relationships between grit and flow in study 2 were as expected, study 3 yielded unexpected results. Specifically, after the original model was found to have a poor fit, the revised model added a direct path from grit to state mindfulness, resulting in a close fit, and also creating a significant, indirect relationship in which state mindfulness mediates the relationship between grit and state flow. As will be discussed in the limitations section, the sample size was relatively small, and given that *p* = 0.06 for the direct effect of grit predicting flow, it is possible that there was insufficient power for detecting this relationship in study 3. While the direct relationship between grit and state flow may be very weak or non-existent, by increasing the sample size, significant results may also be found, as indicated by the results of study 2. Additionally, given the strong relationship between mindfulness and flow (as will be discussed), the underlying mechanisms driving the relationship between grit and flow may be similar to those driving the relationship between grit and state mindfulness. Namely, it was proposed that in alignment with the COR Theory (Hobfoll, 1989), “gritty” individuals have honed their abilities to direct cognitive resources toward deliberate practice (Ericsson et al., 1993) and have developed a resiliency that enables them to persist in their pursuit of flow despite setbacks which occur in the workplace. The results of the current study suggest that this ability for directing cognitive resources and developing resiliency is more useful for experiencing mindfulness, a state in which Dane (2011) suggests “attention is focused on [the] present-moment phenomena…” (p. 1000). These results suggest that the shared variance in these constructs may not allow for significant direct effects from grit to both constructs, and thus grit more strongly influences mindfulness, yet still has an indirect effect on flow.

Concerning indirect effects, support was found in study 3 for flow mediating the relationship between flow metacognition and performance, as well as flow metacognition and engagement. However, support was not found for flow mediating the relationship between flow metacognition and burnout in study 3. Given that flow metacognition entails the “perceptions of the usefulness of flow at work for job performance”, from a theoretical perspective, it makes sense that these beliefs may increase flow and drive the outcomes of performance and engagement, but not burnout. Burnout has more to do with a depletion of personal resources, rather than the allocation of cognitive control toward the utilization of these resources (Costantini, 2022). Therefore, while flow itself can reduce burnout, the belief that flow is useful or not for performance may be theoretically unrelated to whether employees experience burnout.

Furthermore, while flow proneness mediated the relationship between grit and all three outcomes in study 2, grit had no significant indirect effects through state flow on any of the outcomes of interest in study 3. This may be due to a high amount of shared variance between state mindfulness and state flow, resulting in insignificant results. This notion is further supported by the fact that state mindfulness had significant indirect relationships through flow with all three of the outcomes of interest. Additionally, state mindfulness had by far the strongest relationships (both direct and indirect) with flow than either of the other antecedents for all relationships in study 3. These results provide strong theoretical support for the relationship between mindfulness and flow. Specifically, the ability to purposefully exert cognitive control toward experiencing flow at work via state mindFulness is highly effective for experiencing flow at work, lending further support for the CCMWF. The strength of the relationship between state mindfulness and flow at work also suggests that mindfulness interventions may be a ripe starting point for researchers to develop flow interventions at work and may be easy interventions that employees and practitioners can begin implementing immediately.

### Theoretical and practical implications

Overall, these findings have wide-ranging implications for both theory and practice. First, broad support was found for the revised CCMWF. This model answers the call for research expanding upon the nomological network of flow at work and exploring the relationships between flow and metacognitive beliefs (Wilson and Moneta, 2016), flow and mindfulness at work (Ceja and Navarro, 2017; Weintraub and Dust, 2020), flow and grit (Duckworth, 2016), and flow and personal resources (Bakker and van Woerkom, 2017; Demerouti and Mäkikangas, 2017).

These findings also provide support for the notion that flow is a volitional and effortful state (Keller and Bless, 2008), and that underexplored relationships support this notion and account for almost 70% of the variance in flow at the between-person level (*R*^2^ = 0.67, Table 9) and almost 20% of the variance at the within-person level in study 3 (*R*^2^ = 0.17, Table 9). This suggests that not only are employees capable of experiencing flow volitionally, but any examination of flow which focuses solely on environmental factors will likely be missing major elements of what drives flow experience for employees at work. Therefore, practitioners may have more success in fostering flow for employees at work if they focus their efforts on developing flow-inducing competencies in individual employees, rather than attempting to manipulate the work environment to be more conducive to the state. Additionally, the findings suggest that individuals are more than passive agents; they can potentially proactively foster the resources needed for experiencing flow at work on their own, despite the environments in which they work. These results may also inform practice at several touchpoints in the employee lifecycle.

### Limitations and future directions

While the current research did provide wide-ranging implications for theory and practice, as in all studies, several limitations of this research must be discussed. First, while MTurk workers have previously been shown to be relatively equivalent to data obtained from in-person samples, if not more diverse (Casler et al., 2013), a comparison of the study populations and the US economy (Table 5) suggests discrepancies between the current sample and the U.S. population (U.S. Bureau of Labor Statistics, 2016). Therefore, future research should aim to replicate these findings with a more representative sample.

It should also be noted that the measure of performance utilized in these studies was subjective. Therefore, future research should utilize objective performance measures to examine whether flow truly objectively influences performance, and if so, whether this relationship may vary across roles, industries, or different types of performance such as task performance and team performance. A subjective measure was also utilized for flow and mindfulness. As such, future research should employ more objective psychophysiological measures of these states, such as heart rate variability (Tozman et al., 2015) and EEG (Tang et al., 2015), to provide further support for the current findings. Additionally, future research should attempt to measure these states through other potential passive data sources, like behavioral proxies (such as logs generated from software engineering tools) to avoid disrupting flow for study participants (Brown et al., 2023).

The current research provided initial support for a new psychometric tool for measuring metacognitive beliefs about the usefulness of flow for job performance. However, this scale did not measure the metacognitive beliefs about a worker's ability to control when they experience flow as conceptualized by Wilson and Moneta (2016). As such, future research should replicate these findings with the current measure, in addition to a measure of the second flow metacognition factor of beliefs about the ability to control the experience. Future research should also attempt to integrate environmental (i.e., job characteristics) and additional individual difference factors (i.e., conscientiousness) into a broader model. However, a larger sample size would be required for such models, and therefore more participants should be employed in future research.

Furthermore, Weintraub and Dust (2020) suggest that mindfulness and flow are fleeting states which fluctuate throughout the day. Given that study 3 only captured these states three times for a single day, it is only a brief snapshot of what may be occurring for a week, month, and year. Future research should examine these relationships over a longer period using a temporal framework to explore whether true causal or reciprocal relationships exist among these constructs at the within-person level across time. Additionally, while the measures of flow and mindfulness in study 3 were state measures focusing on the moment, the measures of engagement and burnout were broader in their timeframes. As such, variations within the day might not be reflective of variations across other units of time (i.e., days, weeks, months, etc.), if the scope of outcome measures is more consistent with these broader timeframes. Therefore, future research should consider using measures that are more temporally aligned.

Additionally, future research should examine the situational context of these relationships, and whether flow, mindfulness, and other cognitive states are more useful in certain environments or for different types of tasks. This is especially true given that both mindfulness and flow have been shown to have positive and negative consequences (Schüler, 2012; Hafenbrack and Vohs, 2018), which may overlap or be inversely related depending on the situation. Recent research by Reina and Kudesia (2020) also found support for a model which posits that metacognitive beliefs about mindfulness also affect one's ability to self-regulate and allocate cognitive resources toward experiencing state mindfulness. Future research should integrate these metacognitive beliefs into the CCMWF to better understand the mechanisms behind one's ability to experience state mindfulness at work and how this affects the prevalence of flow experience and subsequent downstream outcomes of interest.

Finally, future research should aim to develop and validate interventions for the self-regulation of flow at work. The current study provides evidence that interventions rooted in deliberate practice, mindfulness, and metacognition may be fruitful starting points. Such interventions could be utilized at an individual level (i.e., if an individual wants to increase their own prevalence of flow at work) or implemented at the team or organizational level to drive more wide-ranging results. For example, we echo the calls of other authors to empirically examine the efficacy of mindfulness interventions for fostering flow and encourage collaboration of researchers and practitioners to test this seemingly low-hanging fruit in a randomized control field study. Initial mindfulness intervention research outside of the work domain lends support to this notion (Kaufman et al., 2009; Aherne et al., 2011; Scott-Hamilton and Schutte, 2016; Scott-Hamilton et al., 2016; Jian-Hong et al., 2019; Marty-Dugas et al., 2021). Practitioners could also consider reviewing moments in the workplace where workers remember being in flow as a positive experience with favorable outcomes to foster positive usefulness beliefs for the state where appropriate. Furthermore, organizations and individuals may consider developing the skill of deliberate practice to increase their levels of grit, given that the results of the current study suggest that this individual difference may be indicative of the amount of mindfulness and flow experienced on the job, which subsequently predicts performance, engagement, and burnout. These interventions could be self-administered by individuals, implemented by leaders and organizations via workshops (Costantini et al., 2022), delivered via the utilization of virtual nudges (Weintraub et al., 2021), or by a plethora of other modalities.

## Conclusion

In conclusion, the current research aimed to provide initial empirical support for the Cognitive Control Model of Work-related Flow for three studies. Each of the three studies provides support for the validity of a newly developed measure of flow metacognition aimed at measuring workers' usefulness beliefs about flow for their job performance, and study 2 provided initial support for the CCMWF. While initial results suggested a poor fit for the model in Study 3, a slight revision that added grit predicting state mindfulness at work resulted in robust support for the overall model fit, and medium-to-large effect sizes for the outcome variables of interest. Overall, these results not only suggest that employees are not limited to experiencing flow in accordance with how supportive their workplace is for flow experience but also that they are also capable of experiencing flow on the job through their own motivation and ability to exercise cognitive control toward experiencing flow at work. While limitations were discussed, these results provide implications for theory, practice, and future research directions. Namely, the study expanded the nomological network of flow at work by answering calls for research and integrating understudied antecedents of work-related flow related to the ability to focus concentration of cognitive resources toward experiencing flow at work, as well as the relationship between flow and the outcomes of burnout, engagements, and performance. Researchers and practitioners alike are encouraged to utilize the results of this study as a foundation for exploring a more holistic view of flow at work and as a guide for the development and implementation of interventions aimed at increasing flow on the job.

## Data availability statement

The raw data supporting the conclusions of this article will be made available by the authors, without undue reservation.

## Ethics statement

The studies involving human participants were reviewed and approved by Hofstra University. The patients/participants provided their written informed consent to participate in this study.

## Author contributions

All authors contributed to the conception, design of the study, statistical analysis, and manuscript development.

## Footnotes

^1^After the initial week of collecting data at a rate of $2, a feedback question given at the end of the study indicated that participants felt $2 was insufficient; therefore, after consulting with a faculty advisor, the rate was raised to $2.5 during week 2 of data collection.

## Appendix A

### Flow description

*Flow* is the experience of being “in the zone” that is characterized by an intense and focused concentration on the task at hand, a sense of control over the situation, and a feeling of reward simply from doing the activity itself. People feel they become part of the activity while doing it, lose self-consciousness, and experience time speeding up or slowing down (Csikszentmihalyi and Csikszentmihalyi, 1988).

People have described experiencing flow in the following ways:

“My mind isn't wandering. I am not thinking of something else. I am totally involved in what I am doing. My body feels good. I don't seem to hear anything. The world seems to be cut off from me. I am less aware of myself and my problems.” “I am so involved in what I am doing. I don't see myself as separate from what I am doing.” “My concentration is like breathing. I never think of it. I am really quite oblivious to my surroundings after I really get going. I think that the phone could ring, and the doorbell could ring, or the house burn down or something like that. When I start, I really do shut out the whole world. Once I stop, I can let it back in again.”

## Appendix B

### Flow metacognition measure

*When thinking about the work you perform in your job, to what extent do you agree that regularly experiencing flow would…*

1. be a desirable way to work.^b^
2. enhance your overall job performance.
3. be a useful way to work.^a^
4. help you accomplish your daily goals.
5. be valuable for getting everything done on time.^a^
6. make you an overall more effective worker.

^a^Indicates the item removed from the original measure in study 1 based on the results of confirmatory factor analysis.

^b^Indicates the item was also removed in studies 2 and 3.
